# A Novel Feature Extraction Scheme with Ensemble Coding for Protein–Protein Interaction Prediction

**DOI:** 10.3390/ijms150712731

**Published:** 2014-07-18

**Authors:** Xiuquan Du, Jiaxing Cheng, Tingting Zheng, Zheng Duan, Fulan Qian

**Affiliations:** 1 Key Laboratory of Intelligent Computing and Signal Processing of Ministry of Education, Anhui University, Hefei 230601, China; 2 Institute of Information Engineering, Anhui Xinhua University, Hefei 230088, China; E-Mail: cjx@ahu.edu.cn; 3 School of Computer Science and Technology, Anhui University, Hefei 230601, China; E-Mails: ycduan@gmail.com (Z.D.); qianfulan@126.com (F.Q.); 4 School of Mathematical Science, Anhui University, Hefei 230601, China; E-Mail: tt-zheng@163.com

**Keywords:** protein–protein interaction, random forest, ensemble coding, DX score

## Abstract

Protein–protein interactions (PPIs) play key roles in most cellular processes, such as cell metabolism, immune response, endocrine function, DNA replication, and transcription regulation. PPI prediction is one of the most challenging problems in functional genomics. Although PPI data have been increasing because of the development of high-throughput technologies and computational methods, many problems are still far from being solved. In this study, a novel predictor was designed by using the Random Forest (RF) algorithm with the ensemble coding (EC) method. To reduce computational time, a feature selection method (DX) was adopted to rank the features and search the optimal feature combination. The DXEC method integrates many features and physicochemical/biochemical properties to predict PPIs. On the Gold Yeast dataset, the DXEC method achieves 67.2% overall precision, 80.74% recall, and 70.67% accuracy. On the Silver Yeast dataset, the DXEC method achieves 76.93% precision, 77.98% recall, and 77.27% accuracy. On the human dataset, the prediction accuracy reaches 80% for the DXEC-RF method. We extended the experiment to a bigger and more realistic dataset that maintains 50% recall on the Yeast All dataset and 80% recall on the Human All dataset. These results show that the DXEC method is suitable for performing PPI prediction. The prediction service of the DXEC-RF classifier is available at http://ailab.ahu.edu.cn:8087/DXECPPI/index.jsp.

## 1. Introduction

Protein–protein interactions (PPIs) [1,2] play crucial roles in virtually every biological function. Proteins interact with each other to form protein–protein complexes and perform different biological processes, including metabolism, immune response, endocrine function, and DNA replication [3,4]. Various experimental and computational methods (e.g., two-hybrid systems [5,6], mass spectrometry [7], and protein chip technology [8]) have been developed to detect PPIs. PPIs have generally been studied individually by small-scale biochemical and biophysical experimental techniques. However, these experimental approaches are usually time-consuming and expensive. In recent years, high-throughput biology experimental methods [9,10] have been developed to produce PPIs.

Owing to the drawbacks of experimental methods, significant interest has been placed into the development of computational methods that predict PPIs. These computational methods can be roughly divided into sequence based [11,12,13,14,15,16,17,18,19], structure based [20,21,22,23,24], and function annotation based [25,26,27,28,29] methods with different coding methods, such as autocovariance (AC) [12], local descriptors (LD) [19], conjoint triad (CT) [11], Geary autocorrelation (GAC) [30], Moran autocorrelation (MAC) [31], and normalized Moreau–Broto autocorrelation (NMBAC) [32]. Sequence-based methods have the advantage of not requiring expensive and time-consuming processes to determine protein structures. These methods need to encode only protein sequence pairs to distinguish interaction and non-interaction. AC [12] explains the interactions among the amino acids of protein sub-sequences. AC considers the neighboring effect and enables the discovery of patterns that run through entire sequences. LD [19] is an alignment-free approach and its effectiveness depends largely on the underlying amino acid groups. Each protein is divided into 10 local regions, with each local region containing 3 LD: composition, transition, and distribution. CT [11] considers any 3 continuous amino acids as a unit in the sequence. A number of machine learning methods, including support vector machine [11,12,33], Random Forest (RF) [34], Bayesian network [26,35,36], neural network [37], and conditional random field [38,39], have also been applied to predict PPIs with different features.

In this study, a novel method called DXEC-RF, was developed to predict PPIs. The DXEC method incorporates six coding methods and a feature selection method to construct a classifier. The experiment demonstrates that the ensemble coding (EC) method based on the feature extraction scheme contributes to PPI prediction and is better than other well-known methods using the yeast/human dataset.

## 2. Results and Discussion

### 2.1. Performance Evaluation

PPI prediction is a binary classification problem. In this experiment, precision, recall, accuracy, F-measure, and Matthews correlation coefficient (MCC) were employed to measure the performance of classifiers:

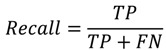
(1)

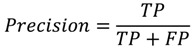
(2)

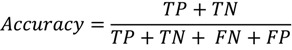
(3)

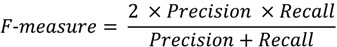
(4)

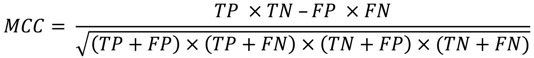
(5)
where TP denotes true interaction pair, TN denotes true non-interaction pair, FP denotes false interaction pair, and FN denotes false non-interaction pair. The ROC (Receiver Operating Characteristic) curve is often used to evaluate classifier performance [40]. A classifier conducts predictions on the basis of a threshold, which generally is defined as 0.5. When the threshold value is changed, new predictions can be obtained and a point can be plotted with the true positive rate (TPR) *versus* the false positive rate (FPR) for different threshold values.

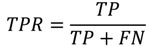
(6)

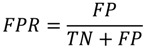
(7)

The area under a curve (AUC) for the ROC curve is also used. When the AUC value of a predictor is larger than the area of other ROC curves, such a predictor is considered better than other predictors.

### 2.2. The DX Result

**Figure 1:**
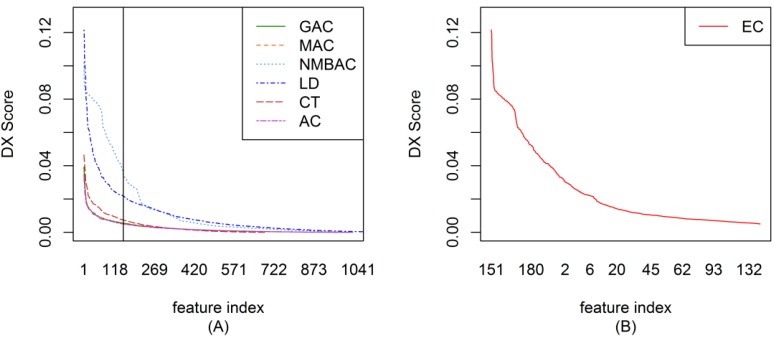
DX score distribution of the different coding methods on the Gold Yeast dataset. (**A**) The result of six methods; (**B**) The result of ensemble coding (EC) method.

**Figure 2:**
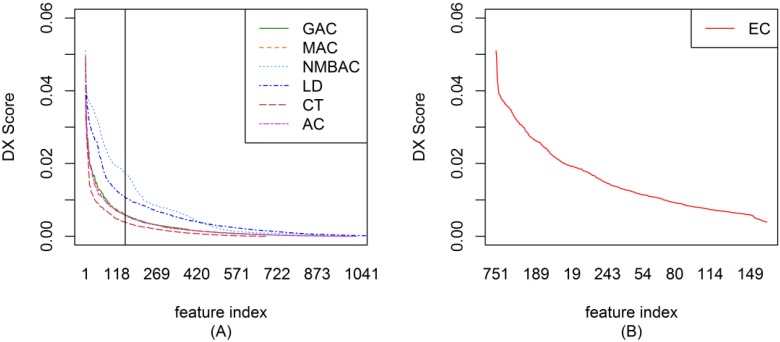
DX score distribution of the different coding methods on the Silver Yeast dataset. (**A**) The result of six methods; (**B**) The result of EC method.

**Figure 3:**
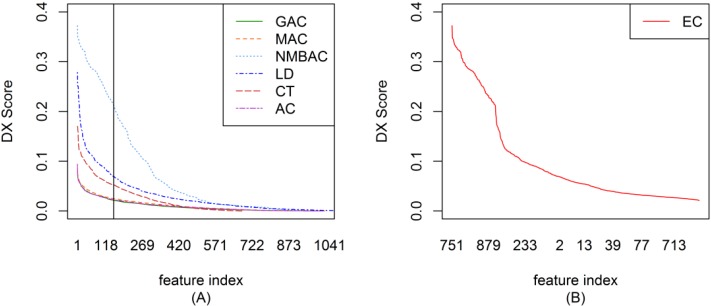
DX score distribution of the different coding methods on the Gold Human dataset. (**A**) The result of six methods; (**B**) The result of EC method.

**Figure 4:**
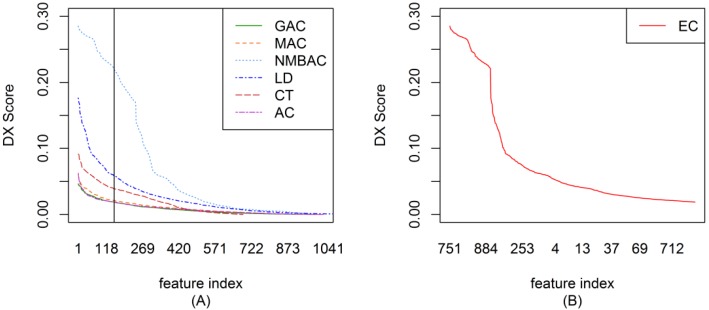
DX score distribution of the different coding methods on the Silver Human dataset. (**A**) The result of six methods; (**B**) The result of EC method.

To ensemble different coding methods, we need to calculate the score of each feature of AC, LD, CT, GAC, MAC, and NMBAC. Figure 1A shows the DX score distribution of each feature of different methods on the Gold Yeast dataset. A larger DX score corresponds to the greater separation of positives and negatives in a feature. To reduce complexity, the top-ranked 150 features from different methods were used to construct EC. The DX method was also adopted in EC to rank each feature. Figure 1B shows the DX score distribution of each feature according to the EC method on the Gold Yeast dataset. The DX score of EC is larger than that of other coding methods on the whole (AC, LD, CT, GAC, MAC, and NMBAC). Figure 2, Figure 3 and Figure 4 show the DX score distribution on the Silver Yeast, Gold Human, and Silver Human datasets, respectively. Table S1 shows the result of using the DX method on the 7 coding methods (AC, LD, CT, GAC, MAC, NMBAC and EC). These tables rank all features according to the DX criteria. The front features denote the features that are more important for PPI prediction in the DX feature table.

### 2.3. Feature Importance Evaluation

**Figure 5:**
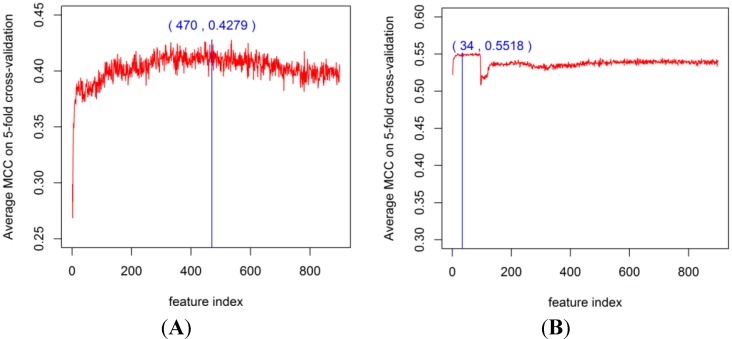
Matthews correlation coefficient (MCC) of DXEC-RF by adding features sequentially on the Yeast dataset. (**A**) The MCC result on the Gold; (**B**) The MCC result on the Silver.

**Figure 6:**
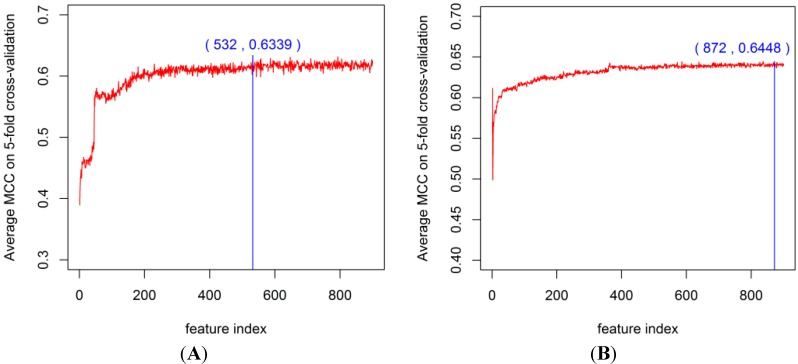
MCC of DXEC-RF by adding features sequentially on the Human dataset. (**A**) The MCC result on the Gold; (**B**) The MCC result on the Silver.

To obtain the best feature space for PPI prediction, we constructed the classifier by using the RF algorithm with the DX feature selection method and EC encoding (DXEC-RF). After ranking each EC feature, incremental feature selection (IFS) [41] was adopted for optimal feature set selection. During the IFS procedure, features were added sequentially from high to low ranking according to the DX score table. The 900 individual predictors corresponding to 900 feature subsets were constructed to train the dataset by using DXEC-RF. The average results of 900 predictors by using 5-fold cross-validation (*i.e.*, the process can be performed 5 times) is presented in Table S2. This feature selection process is illustrated in Figure 5 and Figure 6. The DXEC-RF predictor achieves the highest MCC (*i.e.*, 0.4279) when adopting the top-ranked 470 features on the Gold Yeast dataset (Figure 5A). The DXEC-RF predictor achieves the highest MCC (0.5518) when adopting the top-ranked 34 features on the Silver Yeast dataset (Figure 5B). The DXEC-RF predictor achieves the highest MCC (0.6339) when adopting the top-ranked 532 features on the Gold Yeast dataset (Figure 6A).The DXEC-RF predictor achieves the highest MCC (0.6448) when adopting the top-ranked 872 features on the Silver Yeast dataset (Figure 6B).

### 2.4. Comparison of Prediction Performance by Using Different Methods

**Figure 7:**
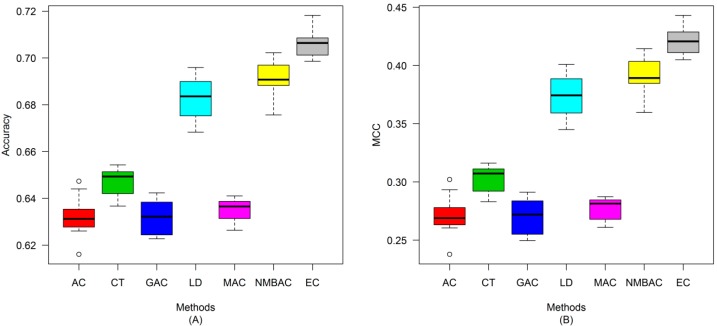
Average accuracy and MCC of different methods on the Gold Yeast dataset with five-fold cross-validation. (**A**) Accuracy; (**B**) MCC.

**Figure 8:**
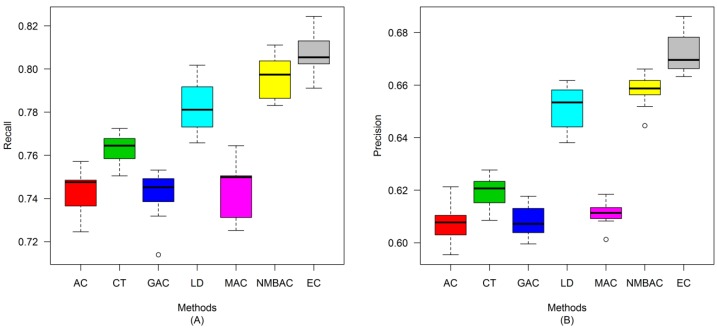
Average recall and precision of different methods on the Gold Yeast dataset with five-fold cross-validation. (**A**) Recall; (**B**) Precision.

**Figure 9:**
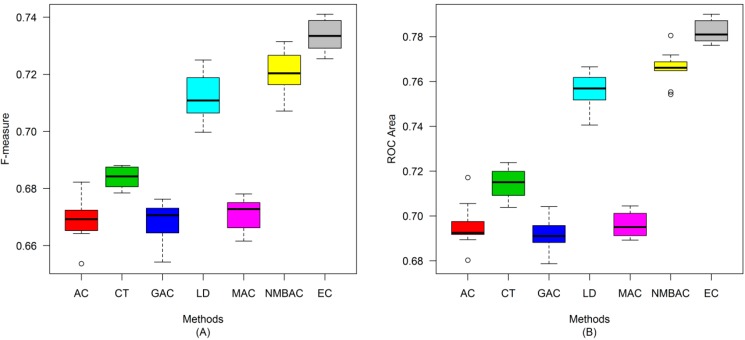
Average F-measure and Receiver Operating Characteristic (ROC) area of different methods on the Gold Yeast dataset with five-fold cross-validation. (**A**) F-measure; (**B**) ROC Area.

**Figure 10:**
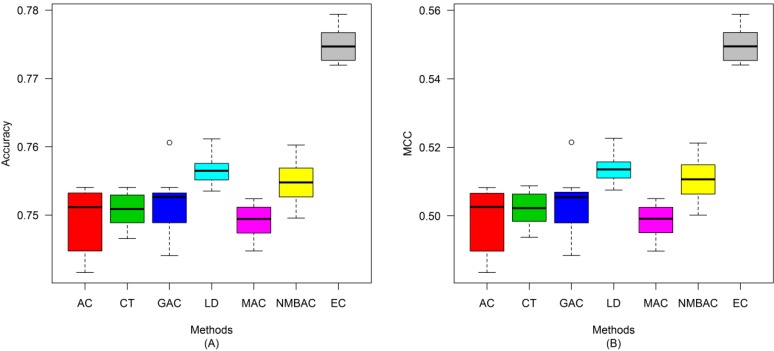
Average accuracy and MCC of different methods on the Silver Yeast dataset with five-fold cross-validation. (**A**) Accuracy; (**B**) MCC.

**Figure 11:**
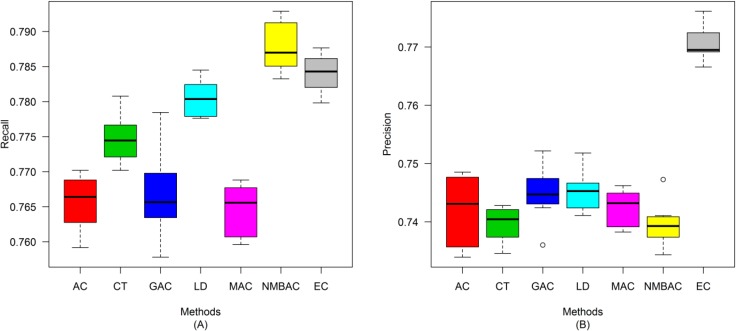
Average Recall and Precision of different methods on the Silver Yeast dataset with five-fold cross-validation. (**A**) Recall; (**B**) Precision.

**Figure 12:**
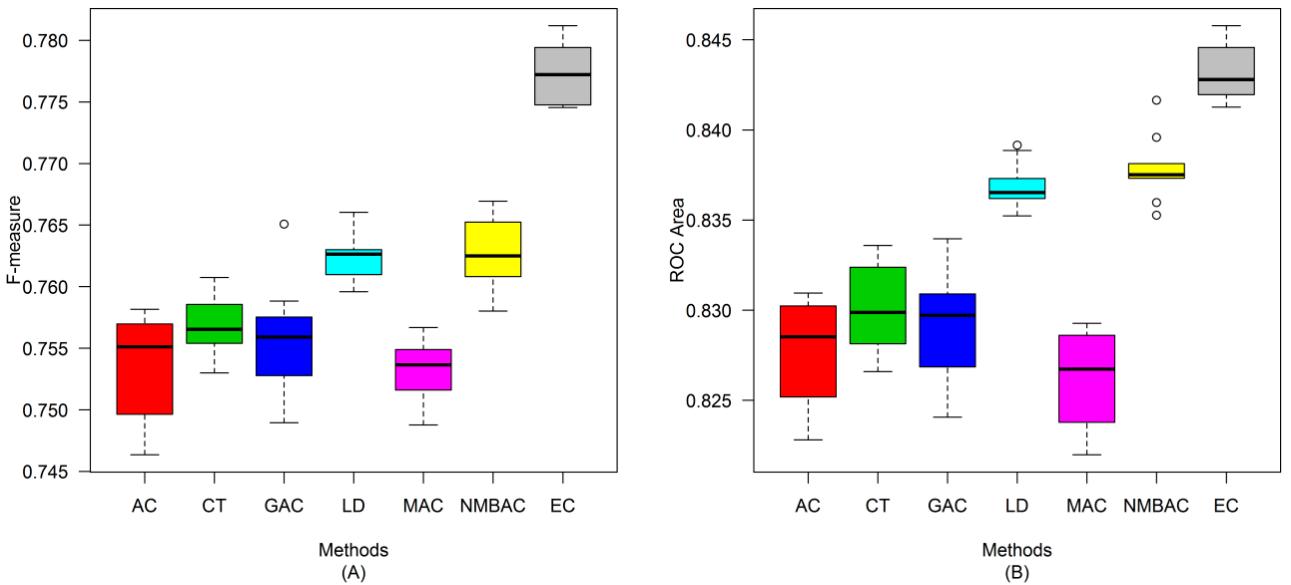
Average F-measure and ROC area of different methods on the Silver Yeast dataset with five-fold cross-validation. (**A**) F-measure; (**B**) ROC Area.

**Figure 13:**
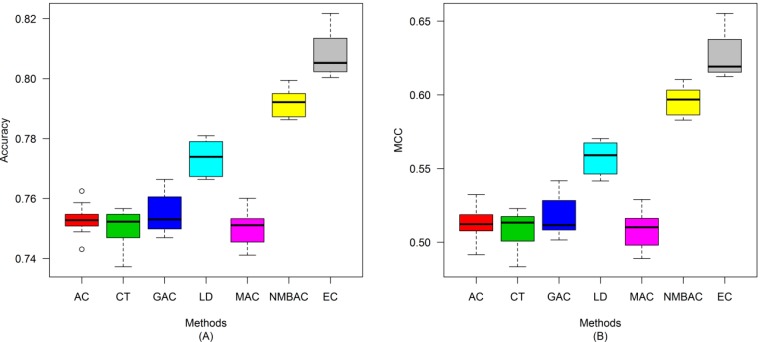
Average accuracy and MCC of different methods on the Gold Human dataset with five-fold cross-validation. (**A**) Accuracy; (**B**) MCC.

**Figure 14:**
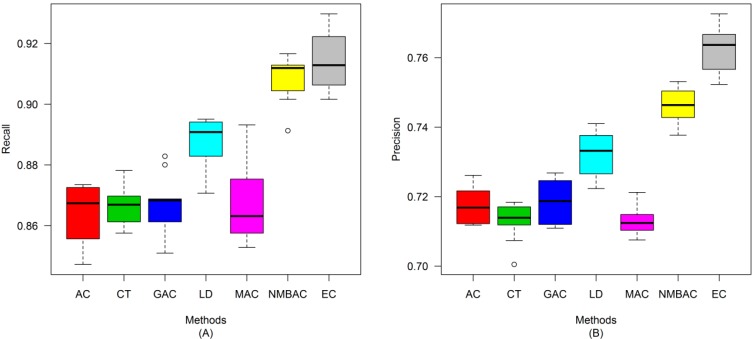
Average recall and precision of different methods on the Gold Human dataset with five-fold cross-validation. (**A**) Recall; (**B**) Precision.

**Figure 15:**
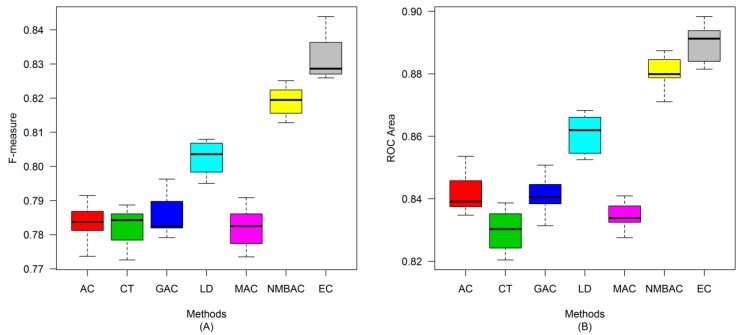
Average F-measure and ROC area of different methods on the Gold Human dataset with five-fold cross-validation. (**A**) F-measure; (**B**) ROC Area.

**Figure 16:**
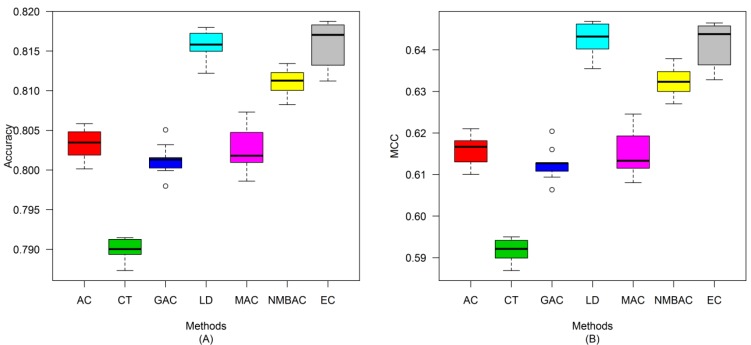
Average accuracy and MCC of different methods on the Silver Human dataset with five-fold cross-validation. (**A**) Accuracy; (**B**) MCC.

**Figure 17:**
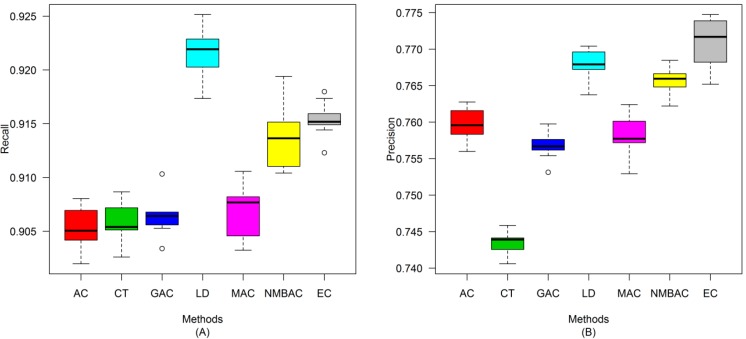
Average recall and precision of different methods on the Silver Human dataset with five-fold cross-validation. (**A**) Recall; (**B**) Precision.

**Figure 18:**
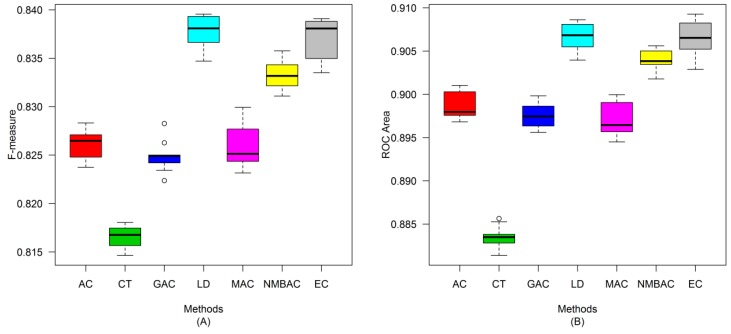
Average F-measure and ROC area of different methods on the Silver Human dataset with five-fold cross-validation. (**A**) F-measure; (**B**) ROC Area.

After the optimal feature subset was confirmed, an experiment was conducted to evaluate the performance of the DXEC method against other coding methods. We performed 7 experiments according to AC, LD, CT, GAC, MAC, NMBAC, and EC by using five-fold cross-validation on the Yeast/Human Gold/Silver datasets; this process can be conducted 10 times. The detailed results are presented in Figure 7, Figure 8, Figure 9, Figure 10, Figure 11, Figure 12, Figure 13, Figure 14, Figure 15, Figure 16, Figure 17 and Figure 18. The EC method obtains the highest accuracy of 70.67%, which is higher by 7.44%, 5.95%, 7.5%, 2.45%, 7.16%, and 1.54% than AC (63.23%), CT (64.72%), GAC (63.17%), LD (68.22%), MAC (63.51%), and NMBAC (69.13%), respectively (Figure 7A). The EC method obtains the highest MCC of 0.422, which is higher by 15.06%, 11.93%, 15.19%, 5%, 14.51%, and 3.06% than AC (0.2714), CT (0.3027), GAC (0.2701), LD (0.372), MAC (0.2769), and NMBAC (0.3914), respectively (Figure 7B). The EC method obtains the highest recall of 80.74%, which is higher than AC (74.32%), CT (76.31%), GAC (74.19%), LD (78.27%), MAC (74.41%), and NMBAC (79.61%) (Figure 8A). The EC method obtains the highest precision of 67.21%, which is higher than the precision of other methods by 1% to 6% (Figure 8B). These results show that the EC method can effectively reduce false PPI prediction.

The EC method scores the highest F-measure of 73.35%, which and is higher than AC (66.9%), CT (61.96%), GAC (60.79%), LD (65.17%), MAC (61.1%), and NMBAC (65.82%), respectively (Figure 9A). The EC method scores the highest ROC area of 0.7824, which is close to the ROC areas of AC (0.6953), CT (0.7146), GAC (0.6918), LD (0.7555), MAC (0.6959), and NMBAC (0.7661), respectively (Figure 9B).

A similar conclusion can be inferred from the data in Figure 10, Figure 11, Figure 12, Figure 13, Figure 14, Figure 15, Figure 16, Figure 17 and Figure 18, excluding the recall of Figure 11A.

### 2.5. Comparison with Other Methods on the All Interaction Datasets

To further assess the performance of the DXEC-RF method, we also tested the ability of trained classifiers on the Yeast/Human All Interaction datasets, which contain all yeast/human protein interactions from the source databases and considers random protein pairs as negatives. Classifiers trained on Gold and Silver Yeast/Human datasets were tested separately. The performance of DXEC-RF is summarized in Table 1, Table 2, Table 3 and Table 4. The other six methods were also implemented on the All Interaction datasets. The DXEC-RF method obtains the highest scores on all parameters, excluding recall (Table 1). The ROC area, MCC, accuracy, and F-measure of DXEC-RF are approximately 1% to 4%, 2% to 6%, 1% to 5%, and 1% to 3% higher than other methods, respectively. Table 2 shows a similar conclusion to that of Table 1. The DXEC-RF obtains a higher recall, F-measure, MCC, and ROC area than other methods (Table 3). When the Silver Human model was used to evaluate the Human All Interaction dataset, the performance of the DXEC-RF is better than other methods (Table 4).

**Figure 19:**
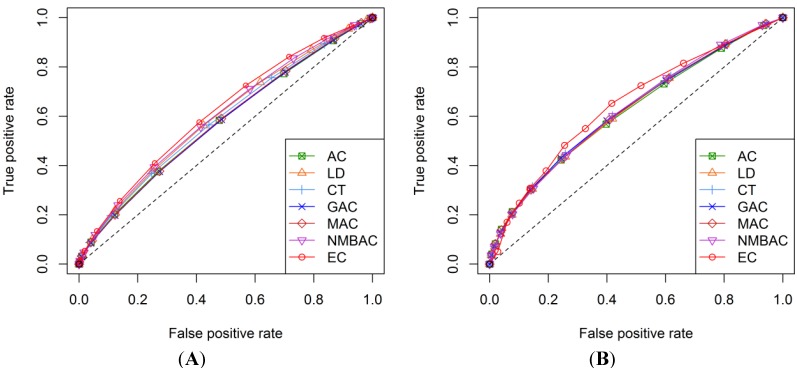
ROC curves of seven methods on the Yeast All dataset. (**A**) Gold Yeast model and (**B**) Silver Yeast model.

**Figure 20:**
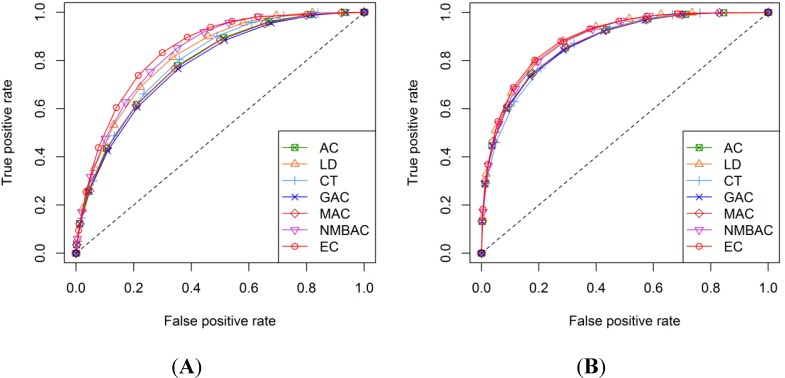
ROC curves of seven methods on the Human All dataset. (**A**) Gold Human model and (**B**) Silver Human model.

Figure 19 and Figure 20 show the ROC curves of each method. As shown in the four graphs, the DXEC-RF method is better than the other methods for both the Yeast and Human All Interaction datasets.

**Table 1:** Performance comparison of seven methods on the Yeast All dataset by using the Gold Yeast model. AC, autocovariance; RF, Random Forest; CT, conjoint triad; LD, local descriptors; MAC, Moran autocorrelation; GAC, Geary autocorrelation; NMBAC, normalized Moreau–Broto autocorrelation; DXEC-RF, the DX feature selection method and ensemble coding (EC) encoding.

Method	Precision	Recall	F-Measure	Accuracy	MCC	ROC Area
AC-RF	0.2897	0.583	0.3871	0.5373	0.091	0.5749
CT-RF	0.2989	0.5645	0.3908	0.5592	0.1058	0.5867
LD-RF	0.3074	0.5625	0.3975	0.5728	0.1207	0.596
MAC-RF	0.2894	0.5905	0.3884	0.534	0.0916	0.576
GAC-RF	0.2877	0.5865	0.386	0.5325	0.0875	0.573
NMBAC-RF	0.31	0.5574	0.3984	0.5783	0.1242	0.6014
DXEC-RF	0.3196	0.5757	0.411	0.5866	0.1445	0.616

**Table 2:** Performance comparison of seven methods on the Yeast All dataset by using the Silver Yeast model.

Method	Precision	Recall	F-Measure	Accuracy	MCC	ROC Area
AC-RF	0.3667	0.4227	0.3927	0.6724	0.1708	0.6222
CT-RF	0.3664	0.4462	0.4024	0.6679	0.1772	0.6289
LD-RF	0.3611	0.4359	0.395	0.6654	0.1679	0.6249
MAC-RF	0.3647	0.4279	0.3938	0.6699	0.17	0.6264
GAC-RF	0.3722	0.4307	0.3993	0.6753	0.1793	0.6272
NMBAC-RF	0.361	0.4427	0.3977	0.664	0.1697	0.6293
DXEC-RF	0.3882	0.4257	0.4061	0.688	0.1955	0.6467

**Table 3:** Performance comparison of seven methods on the Human All dataset by using the Gold Human model.

Method	Precision	Recall	F-Measure	Accuracy	MCC	ROC Area
AC-RF	0.4148	0.7781	0.5411	0.6798	0.367	0.7886
CT-RF	0.4177	0.8025	0.5494	0.6806	0.3816	0.7971
LD-RF	0.437	0.8128	0.5684	0.7005	0.4112	0.8175
MAC-RF	0.4166	0.768	0.5402	0.6827	0.365	0.7857
GAC-RF	0.4092	0.7652	0.5332	0.6749	0.3541	0.7782
NMBAC-RF	0.4839	0.7528	0.5891	0.7452	0.4382	0.8289
DXEC-RF	0.4711	0.8322	0.6016	0.7326	0.4616	0.8414

**Table 4:** Performance comparison of seven methods on the Human All dataset using Silver Human model.

Method	Precision	Recall	F-Measure	Accuracy	MCC	ROC Area
AC-RF	0.4828	0.8528	0.6165	0.7425	0.4851	0.8721
CT-RF	0.4632	0.8705	0.6047	0.7237	0.471	0.8671
LD-RF	0.4972	0.8802	0.6355	0.7549	0.5153	0.8893
MAC-RF	0.4818	0.8549	0.6163	0.7416	0.4851	0.8721
GAC-RF	0.4859	0.8431	0.6165	0.7454	0.4838	0.8704
NMBAC-RF	0.4942	0.8771	0.6322	0.7523	0.5103	0.8846
DXEC-RF	0.5049	0.8809	0.6419	0.7615	0.5242	0.8911

## 3. Materials and Methods

### 3.1. Preparation of Datasets

This study is divided into 3 phases: the feature selection phase, training phase, and testing phase. We used the Yeast and Human protein interaction datasets derived by Indrajit Saha *et al.* [27]. The dataset was composed of 3 datasets extracted from the DIP, MINT, BioGrid, and IntAct databases. We constructed 3 types of datasets, namely, the Gold, Silver, and All Interaction datasets. The Gold dataset contains the PPIs confirmed at least twice by using 2 different experimental methods: the yeast 2-hybrid method and an affinity-based method. The Silver dataset contains PPIs that were confirmed more than once but not necessarily with different experimental methods. The All Interaction dataset was confirmed by using at least one experimental method. On the basis of these datasets, we retained the PPIs with protein sequence lengths larger than 50.

Selecting non-interacting pairs are more complex and important than selecting positive datasets. Random pairs that were not in the positive dataset were used as non-interacting datasets in other studies. In the current study, we created a dataset of negative examples by pairing protein entries randomly selected from UniProtKB. These random pairs were then cross-checked against PPI datasets to remove any true positives. Interacting and non-interacting pairs were labeled as the “positive class” (denote as 1) and “negative class” (denoted as −1), respectively. Table 5 presents the number of instances in each dataset.

### 3.2. Molecular Descriptors

#### 3.2.1. AARC (Amino Acid Residue Change) Features

The AARC (Amino Acid Residue Change) features mainly reflect the amino acids characteristics. These physicochemical properties include hydrophobicity, hydrophilicity, polarity, polarizability, solvation free energy, graph shape index, transfer free energy, amino acid composition, CC in regression analysis, residue accessible surface area in tripeptide, partition coefficient, and formation entropy. These features were also used by Shi *et al.* [18]. The 12 physicochemical properties for each amino acid are shown in Table S3.

**Table 5:** Details of the Gold, Silver, and All Interaction datasets.

Dataset	Organism			
	Yeast		Human	
	Positive	Negative	Positive	Negative
Gold	1503	1503	1067	992
Silver	7267	7244	12680	11877
All interactions	43867	131204	29714	92729

#### 3.2.2. The Features of Amino Acid Factors

Table S4 shows the values of the features of the amino acid factors. Each amino acid residue has different properties that can influence the specificity and diversity of protein structure and function. Atchley *et al.* [42] performed feature analysis on the AAIndex [43] by using the multivariate statistical method and then transformed the AAIndex to five multidimensional and highly interpretable numeric patterns of attribute covariation that reflects polarity, secondary structure, molecular volume, codon diversity, and electrostatic charge. The five numerical pattern scores (denoted as “amino acid factors”) were adopted in the current study to represent the respective properties of each amino acid in a given protein.

### 3.3. Feature Selection (DX)

We know that many features are irrelevant and redundant for a prediction problem. Therefore, a number of feature selection methods were proposed to remove the most irrelevant and redundant features to improve the performance of learning models. We used the DX score [44] to solve this problem. The author of this method adopted the DX score to select the most relevant bigram features. The DX score can assess the discrimination power of a feature in general cases. According to [45], the DX score can be defined as follows:

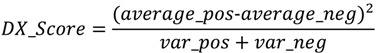
(8)
where average_pos denotes the mean value of the feature in the interaction pairs of the training dataset; average_neg denotes the mean value of the feature in the non-interaction pairs of the training dataset; var_pos and var_neg denote the variance of the feature in the interaction pairs and non-interaction pairs of the training dataset, respectively.

### 3.4. Ensemble Coding Scheme

**Figure 21:**
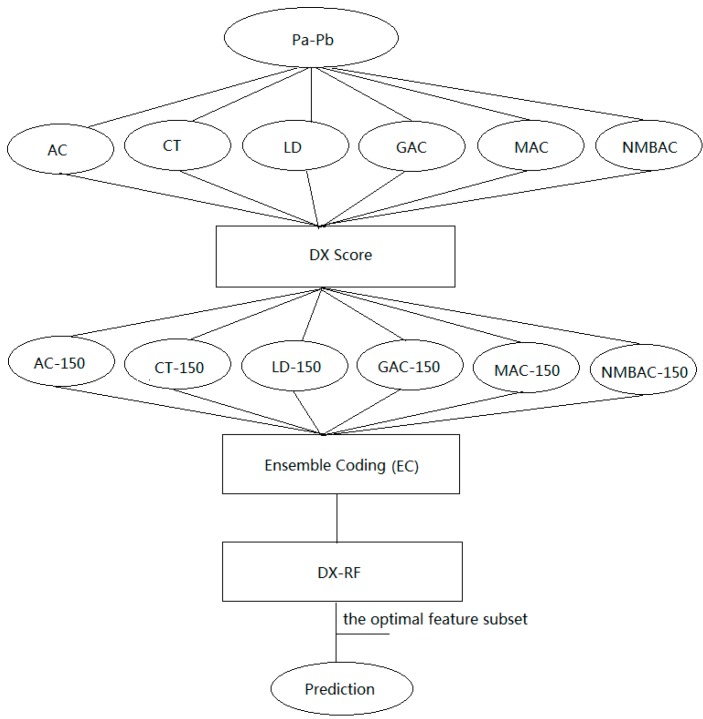
Framework of EC scheme for protein sequence pairs. AC-150 denotes the top-ranked 150 features; the other features are the same as AC-150 and DX-RF. We adopt the RF algorithm to evaluate the importance of each feature with the DX score by adding features sequentially.

The key issue for the sequence-based method in PPI prediction is the manner in which to encode protein sequences by using protein properties. Many studies have proposed effective coding methods, including AC, CT, LD, GAC, MAC, and NMBAC. These methods use the interaction information among amino acids and the amino acid composition according to the protein characteristics. Figure 21 provides the framework of the EC scheme for protein sequence pairs. Take the following protein interaction pair P_a_–P_b_ for example: P_a_, MTASVSNTQNKLNELLDAIRQEF; P_b_, MNPGGEQTI.

First, the sequence P_a_–P_b_ is transformed to six vectors according to the AC, LD, CT, GAC, MAC, and NMBAC methods. AC features describe the level of correlation between two protein sequences in terms of their specific physicochemical property, which are defined on the basis of the distribution of amino acid properties along the sequence.

AC can be computed according to Equation (9).

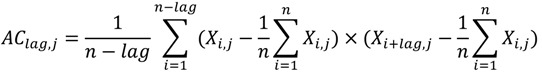
(9)

GAC can be computed according to Equation (10).

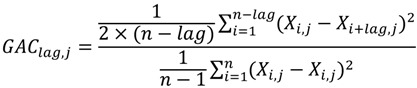
(10)

MAC can be computed according to Equation (11).

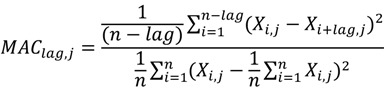
(11)

NMBMAC can be computed according to Equation (12).

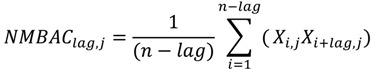
(12)
where *j* represents one descriptor, *i* is the position of the residue in sequence *X*, *n* is the length of the sequence, and *lag* is the distance between a residue and its neighboring residue. In this case, lag is set to 30 for the AC, MAC, GAC, and NMBAC method. In the LD method, each protein is divided into 10 local regions, each of which includes 3 local descriptors: composition, transition, and distribution. The CT method considers any 3 continuous amino acids as a unit in the sequence. The details of such units are provided in the Supplementary Information [11,12,18,30,31,32,46,47,48,49]; Second, the DX method was used to rank each feature (from high to low) for the 6 vectors generated in the first step; Third, given that the dimensions of the AC, LD, CT, GAC, MAC, and NMBAC methods are large, we select the top-ranked 150 features in the 6 coding methods to reduce complexity and combine such features to 1 vector (EC). Fourth, the DX method was adopted to rank each feature in the EC. Fifth, RF was used to evaluate the important of each feature by using 5-fold cross-validation and by adding features sequentially. After the optimal features were confirmed, the model produced by the optimal features was applied to classify the test dataset.

## 4. Conclusions

In this study, a new method was developed for PPI prediction. The EC was constructed by using six coding methods, namely, AC, CT, LD, MAC, GAC, and NMBAC. To extract important features, feature selection (DX score) was used to rank each feature. RF was then adopted to find the optimal feature subset. After the optimal features subset was confirmed, a model was produced on the basis of the optimal feature set and then applied to the Yeast/Human All Interaction datasets. Our results show that the DXEC-RF is more suitable for performing PPI prediction than other methods.

## Supplementary Files

Supplementary File 1
