# Supplementary material for: A Novel Feature Extraction Scheme with Ensemble Coding for Protein–Protein Interaction Prediction
Source: Int J Mol Sci. 2014 Jul 18;15(7):12731–49. doi: 10.3390/ijms150712731 (PMC4139871; doi:10.3390/ijms150712731)
Supplement: Supplementary File 1 [file ijms-15-12731-s001.zip › ijms-56787-supp-to be published/ijms-56787-supplementary.pdf]

Supplementary Information

1. Six Feature Representation Methods

1.1. Auto Covariance (AC)

Auto Covariance (AC) variables describe the relation among residue with a certain number of amino acids (lag) apart in the sequence. Given a protein sequence, the lag is the distance between current residue and its neighboring residue, so this method can take neighboring effect into account. The AC can be computed according to the Equation (1).

$$AC_{lag,j} = \frac{1}{n-lag}\sum_{i=1}^{n-lag}(X_{i,j} - \frac{1}{n}\sum_{i=1}^{n}X_{i,j}) \times (X_{i+lag,j} - \frac{1}{n}\sum_{i=1}^{n}X_{i,j}) \quad (1)$$

where j represents one descriptor, i is the position of the residue in the sequence X, n is the length of the sequence, lag is the distance between residue and its neighboring residue. For detailed descriptions of these descriptors, interested readers could refer to [12].

1.2. Conjoint Triad (CT)

Conjoint triad (CT) regards any three continuous amino acids as a unit in the protein sequence. According to this method, there are 20 amino acid and the dimension of a protein sequence were $20 \times 20 \times 20 = 8000$, it is very large. So, first, the 20 amino acids have been clustered into seven classes according to the dipoles and volumes of the side chains. And thus the dimensions of a protein sequence were dramatically reduced to $7 \times 7 \times 7 = 343$. Then, the descriptors of two proteins were concatenated and a total 686-dimensional vector has been built to represent each protein pair. For detailed descriptions of these autocorrelation descriptors, interested readers could refer to [11].

1.3. Local Descriptor (LD)

Local descriptor (LD) [18] is an alignment-free method. In order to reduce the complexity, we first used the same selection of amino acid grouping according to the CT method. Then each protein is divided into ten local regions of varying length and composition. For each local region, three local descriptors, composition (C), transition (T) and distribution (D), are calculated. C stands for the composition of each amino acid group along a local region. T represents the percentage frequency with which amino acid in one group is followed by amino acid in another group. D characterizes the distribution pattern along the entire region by measuring the location of the first, 25%, 50%, 75% and 100% of residues of a given group. For detailed descriptions of these descriptors, please refer to [18]. Given that the amino acids are divided into seven groups in this instance, the calculation of these descriptors generates 63 attributes in each local region (7 for C, 21 for T and 35 for D). The descriptors for all local regions were combined, resulting in 630 features representing the general characteristics of the protein sequence. Thus, a 1260-dimensional vector has been built to represent each protein pair.

1.4. Autocorrelation (MAC, GAC, NMBAC)

Autocorrelation features describe the level of correlation between two protein sequences in terms of their specific physicochemical property, which are defined based on the distribution of amino acid properties along the sequence. There are 17 amino acid properties used for deriving autocorrelation descriptors as the AC method. Here we use three commonly-used autocorrelation for predicting PPIs, *i.e.*, Geary autocorrelation (GA) [30], Moran autocorrelation (MA) [46,31], and Normalized Moreau-Broto autocorrelation (NA) [32]. For the detailed descriptions of these autocorrelation descriptors, interested readers could refer to [47].

2. Random Forest

In the current study, Random Forest (RF) is adopted as the prediction engine and operated with the default parameters with 5-fold cross-validation. It is an ensemble learning method for classification/regression that consists of a multitude of decision trees at training time. A new prediction sample is located into each of the trees in the forest and each decision tree gives a predicted label. The label of sample with most votes as the predicted label of the random forest. The detailed process can be found in the [48]. In this experiment, Weka [49] software is used to implement the Random Forest algorithm for protein-protein interaction prediction.
